# Transcatheter edge-to-edge repair vs medical therapy in atrial functional mitral regurgitation: a propensity score-based comparison from the OCEAN-Mitral and REVEAL-AFMR registries

**DOI:** 10.1093/eurheartj/ehaf511

**Published:** 2025-08-01

**Authors:** Tomohiro Kaneko, Nobuyuki Kagiyama, Shinya Okazaki, Masashi Amano, Yukio Sato, Yohei Ohno, Masaru Obokata, Kimi Sato, Kojiro Morita, Shunsuke Kubo, Yuki Izumi, Masahiko Asami, Yusuke Enta, Shinichi Shirai, Masaki Izumo, Shingo Mizuno, Yusuke Watanabe, Makoto Amaki, Kazuhisa Kodama, Hisao Otsuki, Toru Naganuma, Hiroki Bota, Masahiro Yamawaki, Hiroshi Ueno, Gaku Nakazawa, Daisuke Hachinohe, Toshiaki Otsuka, Mike Saji, Masanori Yamamoto, Kentaro Hayashida

**Affiliations:** Department of Cardiovascular Biology and Medicine, Juntendo University Graduate School of Medicine, 2-1-1 Hongo, Bunkyo-Ku, Tokyo, Japan; Department of Cardiovascular Biology and Medicine, Juntendo University Graduate School of Medicine, 2-1-1 Hongo, Bunkyo-Ku, Tokyo, Japan; Department of Cardiovascular Biology and Medicine, Juntendo University Graduate School of Medicine, 2-1-1 Hongo, Bunkyo-Ku, Tokyo, Japan; Department of Heart Failure and Transplantation, National Cerebral and Cardiovascular Center, Suita, Japan; Department of Cardiology, St. Marianna University School of Medicine, Kanagawa, Japan; Department of Cardiology, Tokai University School of Medicine, Isehara, Japan; Department of Cardiovascular Medicine, Gunma University Graduate School of Medicine, Maebashi, Japan; Department of Cardiology, Institute of Medicine, University of Tsukuba, Tsukuba, Japan; Department of Nursing Administration and Advanced Clinical Nursing, Division of Health Sciences and Nursing, Graduate School of Medicine, The University of Tokyo, Tokyo, Japan; Department of Cardiology, Kurashiki Central Hospital, Kurashiki, Japan; Department of Cardiology, Sakakibara Heart Institute, Tokyo, Japan; Division of Cardiology, Mitsui Memorial Hospital, Tokyo, Japan; Department of Cardiology, Sendai Kosei Hospital, Sendai, Japan; Department of Cardiology, Kokura Memorial Hospital, Kitakyushu, Japan; Department of Cardiology, St. Marianna University School of Medicine, Kanagawa, Japan; Department of Cardiology, Shonan Kamakura General Hospital, Kamakura, Japan; Department of Cardiology, Teikyo University School of Medicine, Tokyo, Japan; Department of Heart Failure and Transplantation, National Cerebral and Cardiovascular Center, Suita, Japan; Division of Cardiology, Saiseikai Kumamoto Hospital Cardiovascular Center, Kumamoto, Japan; Department of Cardiology, Tokyo Woman's Medical University, Tokyo, Japan; Department of Cardiology, New Tokyo Hospital, Chiba, Japan; Department of Cardiology, Sapporo Higashi Tokushukai Hospital, Sapporo, Japan; Department of Cardiology, Saiseikai Yokohama City Eastern Hospital, Yokohama, Japan; Second Department of Internal Medicine, Toyama University Hospital, Toyama, Japan; Division of Cardiology, Department of Medicine, Kindai University Faculty of Medicine, Osaka, Japan; Division of Cardiology, Sapporo Cardio Vascular Clinic, Sapporo, Japan; Department of Hygiene and Public Health, Nippon Medical School, Tokyo, Japan; Department of Cardiology, Sakakibara Heart Institute, Tokyo, Japan; Division of Cardiovascular Medicine, Department of Internal Medicine, Toho University Faculty of Medicine, Tokyo, Japan; Department of Cardiology, Toyohashi Heart Center, Toyohashi, Japan; Department of Cardiology, Nagoya Heart Center, Nagoya, Japan; Department of Cardiology, Gifu Heart Center, Gifu, Japan; Department of Cardiology, Keio University School of Medicine, Tokyo, Japan

**Keywords:** Atrial functional mitral regurgitation, Transcatheter edge-to-edge repair, Heart failure, Mitral regurgitation

## Abstract

**Background and Aims:**

Atrial functional mitral regurgitation (AFMR) commonly affects elderly and frail individuals. The prognostic impact of transcatheter edge-to-edge repair (TEER) for AFMR has not been investigated.

**Methods:**

Patients with AFMR who underwent TEER were selected from the OCEAN-Mitral registry, and medically managed controls were selected from the REVEAL-AFMR registry, using an identical AFMR definition. The primary endpoint was a composite of all-cause mortality and heart failure hospitalization. The secondary endpoint was all-cause mortality.

**Results:**

A total of 1081 patients (mean age 80.1 ± 8.2 years, 60.5% female) with moderate or severe AFMR were included, of whom 441 underwent TEER and 640 remained on medical treatment. Overlap weighting based on the propensity score yielded well-balanced characteristics (*n* = 441 vs 640; all standardized mean differences <0.01), where TEER was associated with a lower incidence of the primary (hazard ratio [HR] 0.65, 95% confidence interval [CI] 0.43–0.99, *P* = .044) and secondary endpoints (HR 0.58, 95% CI 0.35–0.99, *P* = .044). In an exploratory subgroup analysis, favourable outcomes might be pronounced in patients with ≤mild residual AFMR after TEER, while event rates in those with ≥ moderate residual AFMR were comparable with the medication group. As sensitivity analyses, inverse probability of treatment weighting (*n* = 158 vs 173), propensity score matching (*n* = 104 vs 104), and multivariable Cox regression (*n* = 441 vs. 640) all confirmed favourable associations of TEER with both endpoints.

**Conclusions:**

In real-world data, TEER for patients with moderate or severe AFMR were associated with a lower incidence of adverse events compared with medical treatment.

## Structured Graphical Abstract

**Structured Graphical Abstract:**
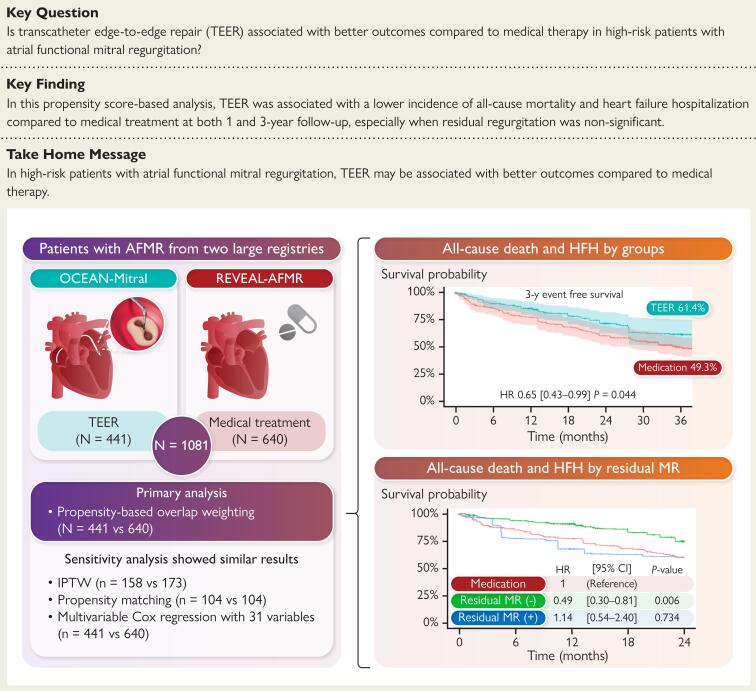
Comparison of patients with atrial functional mitral regurgitation treated with transcatheter edge-to-edge mitral valve repair vs medical therapy. CI, confidence interval; HFH, heart failure hospitalization; HR, hazard ratio; IPTW, inverse probability of treatment weighting; MR, mitral regurgitation; TEER, transcatheter edge-to-edge repair.

**See the editorial comment for this article ‘Transcatheter edge-to-edge repair for atrial functional mitral regurgitation: the final frontier’, by B. McDonaugh *et al.*, https://doi.org/10.1093/eurheartj/ehaf792.**

## Introduction

Mitral regurgitation (MR) represents one of the leading causes of cardiac surgery and is associated with adverse clinical outcomes, including increased morbidity and mortality.^1,2^ Atrial functional mitral regurgitation (AFMR) is an emerging type of MR, recently recognized as a distinct entity characterized primarily by annular dilatation secondary to left atrial enlargement, and its incidence rises significantly with advancing age.^3–5^ Surgical intervention is the primary treatment option for AFMR because of its capability to concurrently address other concomitant cardiac conditions, including tricuspid regurgitation (TR), and potential benefits in terms of improving prognosis.^6–8^ However, many patients with AFMR are elderly and frail, rendering them unsuitable candidates for conventional surgery.^9^

Transcatheter edge-to-edge repair (TEER) has emerged as a valuable therapeutic option for patients with MR who are at high surgical risk.^10,11^ In particular, large randomized controlled trials (RCTs) have demonstrated the efficacy of TEER in reducing mortality and heart failure hospitalization in patients with ventricular functional mitral regurgitation (VFMR).^12,13^ However, AFMR and VFMR differ fundamentally in their underlying mechanisms, suggesting potentially distinct optimal treatment strategies.^3^ For instance, while surgical intervention may not consistently yield favourable outcomes in VFMR, it may be beneficial in AFMR.^14,15^ Although small single-arm observational studies have reported reduced MR severity and improved quality of life following TEER in patients with AFMR,^7,16,17^ whether these findings translate into improved prognosis by TEER for AFMR remains a critical question that warrants thorough investigation.

To date, no studies, including RCTs or large-scale observational studies have specifically investigated the prognostic impact of TEER compared with medical therapy in AFMR. Therefore, we conducted a comprehensive investigation into the association between TEER and prognosis in patients with AFMR by integrating data from two large-scale Japanese AFMR registries and using appropriate statistical methods.

## Methods

### Study design

We utilized the following two registries to compare patients who underwent TEER with those who remained under medical treatment; Supplementary data online, *Figure S1* shows the patient inclusion chart, and full details of the Methods are available in the Supplementary Materials. The Optimized Catheter Valvular Intervention (OCEAN-Mitral) registry is an ongoing, prospective, multicentre registry involving 21 centres in Japan.^18^ This registry, led by physicians to evaluate the safety and efficacy of TEER for MR, has enrolled consecutive adult patients (≥20 years old) with MR who underwent TEER from April 2018 to June 2023. TEER in this cohort was exclusively performed using the MitraClip device (Abbott, Santa Clara, CA, USA). The REVEAL-AFMR study was also a physician-initiated study to investigate the real-world characteristics and prognosis of patients with AFMR. This registry included consecutive adult patients (≥20 years old) with moderate or severe AFMR who underwent transthoracic echocardiography between January and December 2019 at 26 Japanese centres regardless of their treatment plan. The design, population, and primary results of these registries were previously published elsewhere.^7,18^

Since OCEAN-Mitral and REVEAL-AFMR had different inclusion and exclusion criteria, we selected patients with moderate or severe AFMR who met the overlapping criteria of both registries to ensure a comparable study population. From each registry, we included patients with AFMR defined as MR in the absence of organic mitral valve disease, with normal left ventricular size and function (left ventricular end-diastolic volume <150 mL, end-diastolic diameter <55 mm, and ejection fraction ≥50%) and a dilated left atrium (left atrium volume index, ≥ 38 mL/m^2^ for men and ≥41 mL/m^2^ for women when available, or, alternatively, left atrial diameter ≥40 mm in men and ≥38 mm in women) for the present analysis.^4,7,19^ Patients who underwent surgical intervention were excluded from the REVEAL-AFMR registry. Furthermore, since there is partial overlap in participating centres between the two registries, some patients who underwent TEER enrolled in the REVEAL-AFMR registry might also be included in the OCEAN-Mitral registry. Therefore, 30 patients who underwent TEER in the REVEAL-AFMR registry were excluded to avoid potential duplication and associated bias. Patients with missing data in the 31 baseline covariates used to estimate the propensity score (see Supplementary data online, *Table S1*) were excluded.

### Endpoints

The primary endpoint was a composite of all-cause mortality and heart failure hospitalization, and the secondary endpoint was all-cause mortality.

### Propensity score-based overlap weighting

Baseline characteristics differed substantially between the TEER and medication groups. To address this imbalance and to estimate the association between TEER and clinical outcomes, we employed several causal inference approaches.

As the primary analysis, we used propensity score-based overlap weighting, which assigns weights to each patient based on the probability of receiving the observed treatment given their baseline characteristics. In the balanced cohort created by overlap weighting, we compared the incidence of the primary and secondary endpoints. After overlap weighting, Kaplan–Meier curves were constructed to visualize event rates in the TEER and the medication groups. Cox proportional hazards models were also constructed to assess the hazard ratio (HR) of the treatment with TEER for the endpoints. Similarly, logistic regression models were employed to assess the odds ratios (OR) of the treatment with 1, 2, and 3 year incidence of the endpoints. To identify subgroups in whom the association between TEER and clinical endpoints might be stronger, pre-specified subgroup analyses for age, sex, MR severity, TR severity, paroxysmal atrial fibrillation, left atrial volume index, and left ventricular end-diastolic diameter were performed to test for interactions.

### Sensitivity analysis

#### Propensity score-based analysis

As a sensitivity analysis, we also performed inverse probability of treatment weighting (IPTW) using the same propensity score as in the overlap weighting.

As another sensitivity analysis, we performed 1:1 greedy propensity score matching using the same covariates as in the overlap weighting, with a caliper width of 0.2 standard deviations (SDs) of the logit of the propensity score. This picks up the same number of patients from each group and creates pairs of patients with similar characteristics.

For patient populations created by these methods, we performed Kaplan–Meier analysis and Cox and logistic regression analyses as in the overlap weighting.

#### Multivariable Cox proportional hazard models

In addition to the propensity score-based analyses, we conducted a traditional multivariable Cox proportional hazard model to estimate the HRs of the treatment with TEER for the primary and secondary endpoints after adjusting for the same covariates as in the propensity score model.

### Statistical analysis

Categorical and continuous variables are presented as numbers (percentages) and means  ± SDs or medians (interquartile range [IQR]), respectively. Differences in baseline characteristics between groups in the unweighted cohort were examined using Welch’s *t*-test, Wilcoxon rank-sum test, or χ^2^ test. To evaluate potential non-linear interactions between baseline left atrial size and treatment group on the primary endpoint, Cox proportional hazards models were utilized. These models included interaction terms between treatment group (TEER vs. medication) and restricted cubic spline functions with three knots representing left atrial volume index and diameter, respectively. The models allowed for estimation and visualization of the HR for the primary endpoint, comparing TEER with the medication reference group, across the continuous range of baseline left atrial sizes.

## Results

### Study population and baseline characteristics

A total of 441 patients who underwent TEER in the OCEAN-Mitral registry and 640 medically treated patients in the REVEAL-AFMR registry were included (see Supplementary data online, *Figure S1*). The baseline characteristics of these patients (unweighted) are shown on the left side of *Table 1*. Patients in the TEER group were older, had more comorbidities, and exhibited more severe heart failure symptoms than those in the medication group. The main medications used are reported in Supplementary data online, *Table S2*. Echocardiographic findings showed that the TEER group had larger left atrial and left ventricular dimensions, slightly lower left ventricular ejection fraction, and more severe MR. Effective regurgitant orifice areas (*n* = 671, 37.9% missing; 0.32 ± 0.15 vs. 0.23 ± 0.08 mm^2^, *P* < .001) and regurgitant volumes (*n* = 605, 44.0% missing; 51 ± 22 vs. 39 ± 14 mL, *P* < .001) were significantly greater in the TEER group.

**Table 1:** Baseline characteristics

	Original baseline characteristics			Overlap-weighted characteristics		
	TEER	Medication	*P*-value	TEER	Medication	SMD
**N**	441	640		441	640	
***Clinical characteristics***						
**Age, years**	82 ± 7	79 ± 9	<.001	81.7	81.7	0
**Male**	180 (40.8%)	247 (38.6%)	.502	36.3%	36.3%	0
**Body surface area, m^2^**	1.47 ± 0.18	1.51 ± 0.18	<.001	1.47	1.47	0
**NYHA (III or IV)**	253 (57.4%)	58 (9.1%)	<.001	28.0%	28.0%	0
***Past medical history***						
**Hypertension**	313 (71.0%)	530 (82.8%)	<.001	76.8%	76.8%	0
**Diabetes mellites**	68 (15.4%)	100 (15.6%)	.995	18.0%	18.0%	0
**Prior symptomatic stroke**	55 (12.5%)	30 (4.7%)	<.001	5.9%	5.9%	0
**Cancer**	68 (15.4%)	137 (21.4%)	.017	17.3%	17.3%	0
**Prior HF admission**	353 (80.0%)	157 (24.5%)	<.001	58.4%	58.4%	0
**Type of atrial fibrillation**						
** Paroxysmal**	78 (17.7%)	106 (16.6%)	.688	15.9%	15.9%	0
** Permanent**	293 (66.4%)	375 (58.6%)	.011	65.1%	65.1%	0
**Coronary artery disease**	96 (21.8%)	89 (13.9%)	<.001	18.8%	18.8%	0
**COPD**	36 (8.2%)	34 (5.3%)	.081	7.1%	7.1%	0
**History of smoking**	127 (28.8%)	199 (31.1%)	.459	28.5%	28.5%	0
***Prescription***						
**Loop diuretics**	371 (84.1%)	323 (50.5%)	<.001	73.4%	73.4%	0
**Tolvaptan**	175 (39.7%)	56 (8.8%)	<.001	21.5%	21.5%	0
**Beta-blocker**	308 (69.8%)	318 (49.7%)	<.001	64.9%	64.9%	0
**MRA**	217 (49.2%)	149 (23.3%)	<.001	42.6%	42.6%	0
***Laboratory data***						
**Haemoglobin, g/dL**	11.16 ± 1.68	11.90 ± 2.07	<.001	11.4	11.4	0
**Creatinine, mg/dL**	1.48 ± 1.04	1.20 ± 1.10	<.001	1.40	1.40	0
**eGFR, mL/min/1.73 m^2^**	39 ± 18	64 ± 24	<.001	48.5	48.5	0
***Echocardiography***						
**LVDd, mm**	49.3 ± 6.8	46.3 ± 4.9	<.001	46.9	46.9	0
**LVDs, mm**	32.8 ± 6.0	30.5 ± 4.6	<.001	31.0	31.0	0
**LVEF, %**	60 ± 6	62 ± 6	<.001	60.8	60.8	0
**LAVI, mL/m^2^**	111 ± 75	81 ± 49	<.001	94.6	94.6	0
**MR grade at rest**			<.001			0
** Moderate**	64 (14.5%)	522 (81.6%)		42.6%	42.6%	
** Moderate–severe**	158 (35.8%)	77 (12.0%)		32.8%	32.8%	
** Severe**	219 (49.7%)	41 (6.4%)		24.6%	24.6%	
**TR grade at rest**			<.001			0
** Moderate**	147 (33.3%)	239 (37.3%)		34.5%	34.5%	
** Moderate–severe**	73 (16.6%)	42 (6.6%)		11.9%	11.9%	
** Severe**	14 (3.2%)	76 (11.9%)		9.3%	9.3%	
**Moderate or severe AS**	18 (4.1%)	74 (11.6%)	<.001	7.9%	7.9%	0

Abbreviations: AS, aortic stenosis; COPD, chronic obstructive pulmonary disease; eGFR, estimated glomerular filtration rate; HF, heart failure; LAVI, left atrial volume index; LVDd, left ventricular end-diastolic diameter; LVDs, left ventricular end-systolic diameter; LVEF, left ventricular ejection fraction; MR, mitral regurgitation; MRA, mineralocorticoid receptor antagonist; NYHA, New York Heart Association; TEER, transcatheter edge-to-edge repair; TR, tricuspid regurgitation.

In the TEER group, acute procedural success was achieved in 420 of 441 patients (95.2%). Only 13 of 441 patients (2.9%) experienced device-related complications (eight leaflet tears and five single-leaflet device attachments). At discharge, residual MR was mild or less in 343 patients (78.3%), moderate in 83 (18.9%), and moderate to severe or severe in 12 (2.8%).

### Unweighted outcomes and follow-up

Over a median follow-up of 754 (IQR 342–1102) days, a total of 320 patients experienced the primary endpoint and 222 experienced the secondary endpoint in the combined unweighted cohort. Kaplan–Meier curves shown in Supplementary data online, *Figure S2* demonstrated a higher incidence of both the primary and secondary endpoints in the TEER group compared with the medication group.

### Overlap weighted cohort

#### Patient balance after weighting

A propensity score-based overlap weighting method was applied to balance the TEER and the medication groups. As shown on the right side of *Table 1*, the two groups became well balanced while retaining the same number of original patients (441 and 640 in the TEER and the medication groups, respectively). After weighting, demographic characteristics, comorbidities, medications, and cardiac and laboratory parameters were similar between groups. The mean age was 81.7 years, 28% had severe heart failure symptoms (New York Heart Association [NYHA] class III or IV), and 42.6% had moderate MR at rest. Effective regurgitant orifice areas and regurgitant volumes were not used for overlap weighting because of significant missing values; however, these values were similar after weighting (effective regurgitant orifice area 0.26 vs. 0.29 mm^2^, *P* = .127; regurgitant volume 43.5 vs 49.7 mL, *P* = .063).

#### Primary and secondary endpoints in the weighted cohort

**Figure 1:**
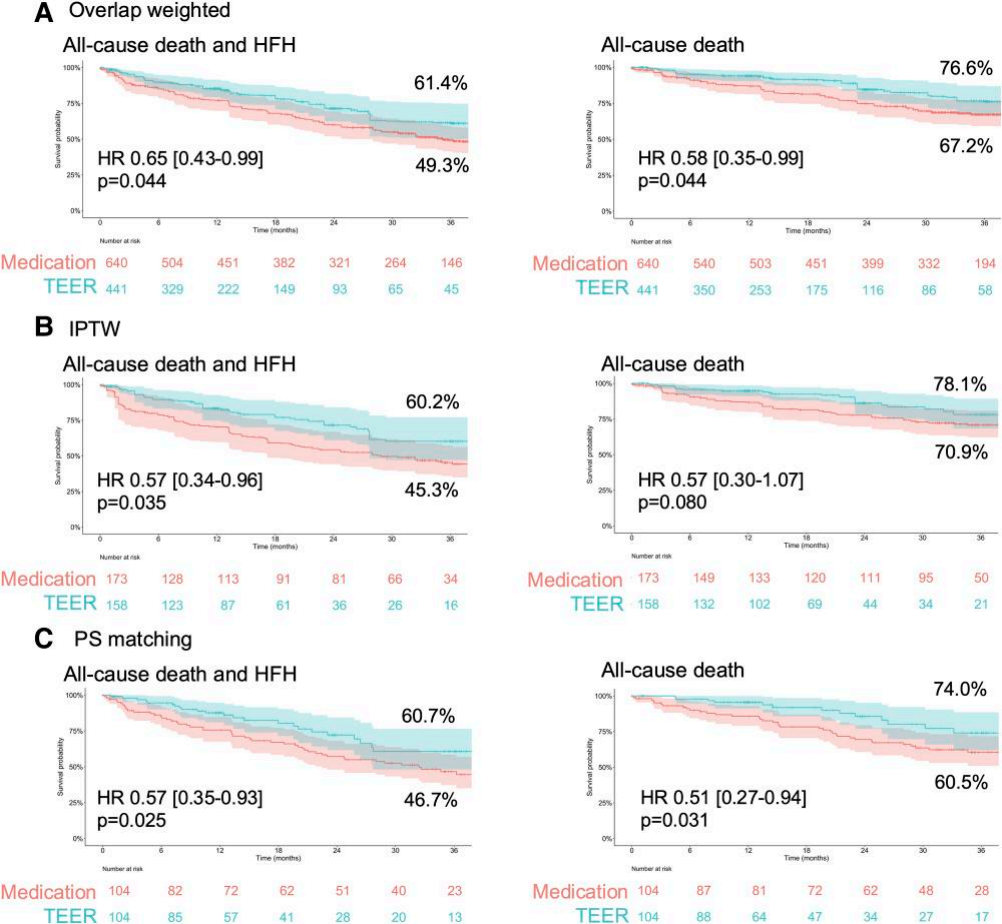
Adjusted Kaplan–Meier curves for both endpoints. Adjusted Kaplan–Meier curves using overlap weighting for the composite endpoint of all-cause mortality and heart failure hospitalization (*A*, left) and all-cause death (*A*, right). Adjusted Kaplan-Meier curves using IPTW for the composite endpoint of all-cause mortality and heart failure hospitalization (*B*, left) and all-cause death (*B*, right). Adjusted Kaplan–Meier curves for 1:1 propensity score matching for the composite endpoint of all-cause mortality and heart failure hospitalization (*C*, left) and all-cause death (*C*, right). Event-free survival estimates with standard errors are shown for 3 years. The TEER-treated group (blue) consistently demonstrate better outcomes compared with the medically treated group (red). HFH, heart failure hospitalization; HR, hazard ratio; IPTW, inverse probability of treatment weighting; PS, propensity score; TEER, transcatheter edge-to-edge repair

Kaplan–Meier curves showed a lower incidence of both primary and secondary endpoints in the TEER group (*Figure 1A*). Cox proportional hazards analysis confirmed that TEER was associated with a lower risk of the primary (HR 0.65, 95% confidence interval [CI] 0.43–0.99, *P* = .044) and secondary endpoints (HR 0.58, 95% CI 0.35–0.99, *P* = .044).

The 1, 2, and 3 year incidence of the primary endpoint was consistently lower in the TEER group, with corresponding ORs and 95% CIs shown in *Table 2*. Similarly, the incidence of the secondary endpoint at 1, 2, and 3 years was lower in the TEER group (see Supplementary data online, *Table S3*).

**Table 2:** Regression analysis for the primary endpoints at 1, 2, and 3 years

		Estimated incidence of the primary endpoint, %		OR	95% CI	*P*-value
		TEER	Medication			
**Overlap weighting**	1 year	12.3	21.7	0.51	0.27–0.96	.036
	2 years	18.4	36.6	0.39	0.23–0.67	.001
	3 years	21.0	44.3	0.33	0.20–0.56	<.001
**IPTW**	1 year	14.4	28.7	0.42	0.18–0.96	.040
	2 years	20.3	43.1	0.34	0.17–0.66	.002
	3 years	23.5	49.9	0.31	0.16–0.59	<0.001
**PS matching**	1 year	10.6	23.1	0.39	0.18–0.84	.018
	2 years	18.3	39.4	0.34	0.18–0.64	.001
	3 years	22.1	47.1	0.32	0.17–0.58	<.001
**Unadjusted analysis**	1 year	20.2	11.9	1.88	1.34–2.62	<.001
	2 years	27.4	20.2	1.50	1.13–1.99	.005
	3 years	31.1	25.2	1.34	1.02–1.76	.033

Abbreviations: CI, confidence interval; IPTW, inverse probability of treatment weighting; OR, odds ratio; PS, propensity score; TEER, transcatheter edge-to-edge repair.

In the weighted cohort, baseline NYHA class distribution was similar between the two groups (NYHA class III, 23.4%; NYHA class IV, 4.6%; *P* > .999). At 1 year follow-up, a more favourable NYHA class distribution was observed in the TEER group (NYHA class III, 0.6%; NYHA class IV, 0.0%) compared with the medication group (NYHA class III, 13.2%; NYHA class IV, 4.5%; *P* < .001) (see Supplementary data online, *Figure S3*).

#### Residual MR and clinical outcomes

**Figure 2:**
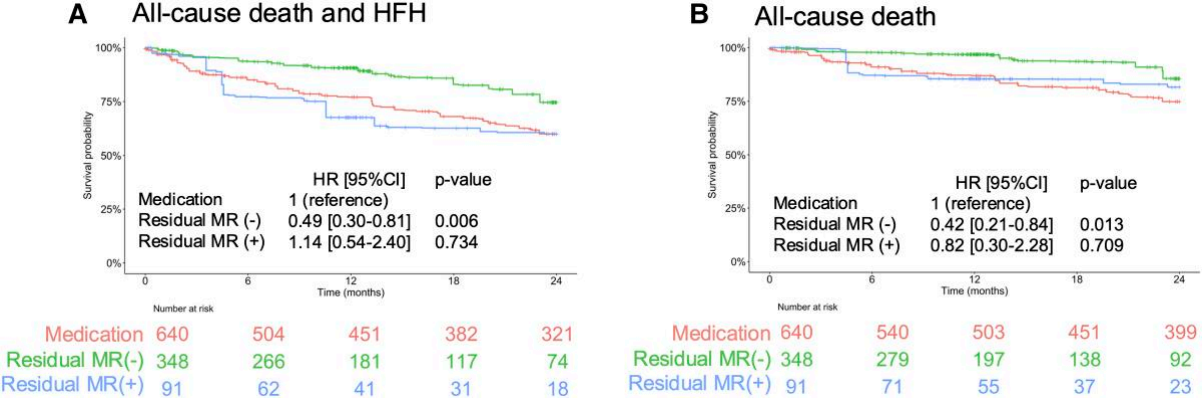
Adjusted Kaplan–Meier curves stratified by residual MR severity at discharge. Adjusted Kaplan–Meier curves stratified by residual MR severity at discharge in the TEER-treated group and the medically treated group for the composite endpoint of all-cause mortality and heart failure hospitalization (*A*) and all-cause death (*B*). Patients with mild or less residual MR (green) show significantly better outcomes compared with those with moderate or severe residual MR (blue) and the medically treated group (red). CI, confidence interval; HFH, heart failure hospitalization; HR, hazard ratio; MR, mitral regurgitation

Within the overlap weighted population, we further examined the impact of residual MR at discharge. Kaplan–Meier curves stratified by the presence or absence of moderate or greater residual MR were compared with the medication group (*Figure 2*). Cox proportional hazard models indicated that patients with mild or less residual MR had a better prognosis compared with the medication group (HR 0.49, 95% CI 0.30–0.81, *P* = .006 for the primary endpoint; HR 0.42, 95% CI 0.21–0.84, *P* = .013 for the secondary endpoint). Among patients with moderate or severe residual MR, event rates were similar to those in the medical therapy group, with HRs of 1.14 (95% CI 0.54–2.40, *P* = .734) for the primary endpoint and 0.82 (95% CI 0.30–2.28, *P* = .709) for the secondary endpoint. A similar pattern was observed when limiting the analysis to patients with moderate residual MR (HR 1.29, 95% CI 0.61–2.74, *P* = .511 for the primary endpoint; HR 0.91, 95% CI 0.32–2.61, *P* = .863 for the secondary endpoint).

#### Exploratory subgroup analysis

**Figure 3:**
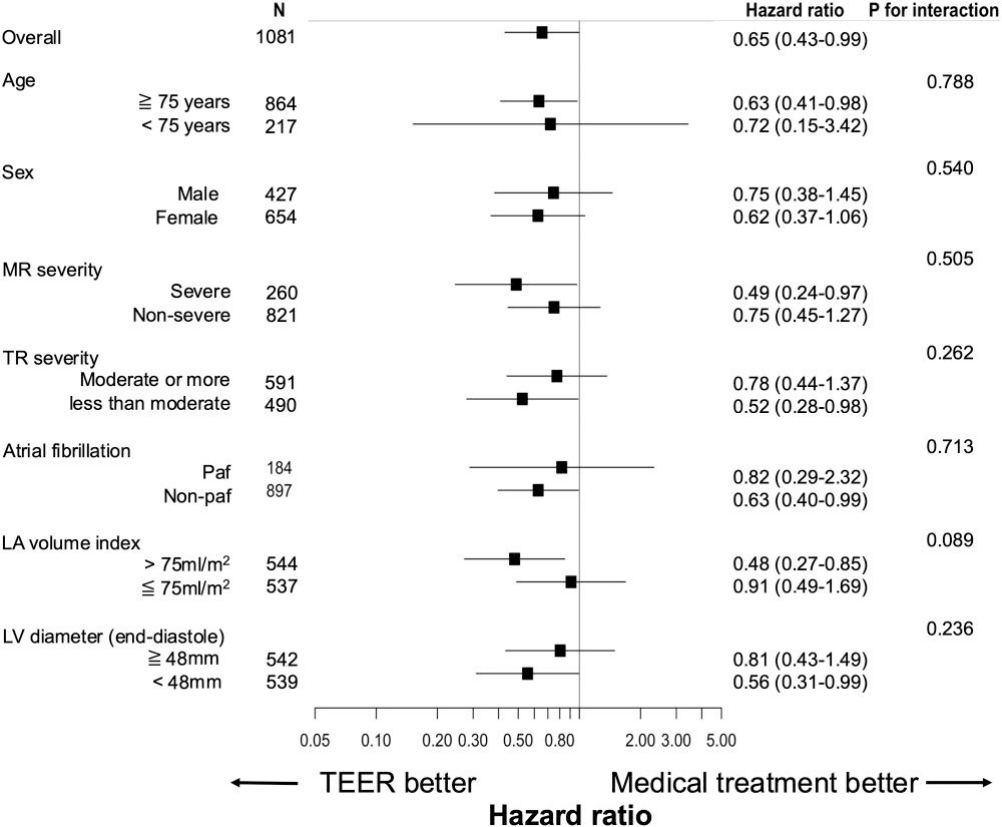
Forest plot of subgroup analysis for all-cause mortality and heart failure hospitalization. Subgroup analyses showing HR with 95% CI for the composite endpoint of all-cause mortality and heart failure hospitalization in TEER-treated vs. medically treated groups. Subgroups include age, sex, MR severity, severity of tricuspid regurgitation, left atrial volume index, and left ventricular diameter. *P*-values for interaction are provided for each subgroup. LA, left atrium; LV, left ventricle; MR, mitral regurgitation; Paf, paroxysmal atrial fibrillation; TR, tricuspid regurgitation

In the weighted cohort, subgroup analyses were conducted to investigate potential associations with clinical outcomes after TEER. Age, sex, severity of MR, severity of TR, paroxysmal atrial fibrillation, left atrial volume index, and left ventricular diameter were not associated with the primary endpoints (*Figure 3*).

Restricted cubic spline analyses indicated that the HR for TEER progressively decreased as left atrial size increased from smaller values up to a certain point. However, this trend plateaued, and the HR potentially increased, with marked left atrial enlargement (approximately beyond a left atrial volume index of 100 mL/m^2^ or a diameter of 60 mm), suggesting attenuation or reversal of TEER's relative benefit at extreme left atrial dimensions (see Supplementary data online, *Figure S4*).

In addition, we examined whether the results were consistent regardless of the presence of significant aortic valve disease. After excluding 101 patients with ≥ moderate aortic stenosis and ≥ moderate to severe aortic regurgitation, overlap weighting analysis yielded similar HRs for the primary and secondary outcomes between the TEER and medication groups (HR 0.66, 95% CI 0.43–1.03, *P* = .064 for the primary outcome; HR 0.61, 95% CI 0.35–1.07, *P* = .088 for the secondary outcome), although these results did not reach statistical significance due to the reduced number of patients.

### Sensitivity analyses for the primary and secondary outcomes

The IPTW analysis, using a propensity score estimated with the same covariates as in the primary analysis, yielded similar results. To extract the optimal subset sample for the most accurate estimation of the average treatment effect, the 750 cases with extreme propensity scores were excluded, leaving 331 patients for analysis (158 in the TEER group and 173 in the medication group). Balance check showed that the standardized mean difference was < 0.10 for most parameters with two exceptions of 0.108 for NYHA class and 0.104 for aortic stenosis (see Supplementary data online, *Table S4*). The adjusted Kaplan–Meier curve shown in *Figure 1B* and Cox models demonstrated that the TEER group had a lower incidence of the primary endpoints. (HR 0.57, 95% CI 0.34–0.96, *P* = .035). A similar trend favouring TEER was observed for the secondary endpoint (HR 0.57, 95% CI 0.30–1.07, *P* = .080), as shown in *Figure 1B*. The 1, 2, and 3 year incidence of the primary and secondary endpoint was consistently lower in the TEER group, as summarized in *Table 2* and Supplementary data online, *Table S3*.

Propensity score matching yielded, using the same covariates as in the primary analysis, 104 pairs (208 patients in total) of patients with similar baseline characteristics, as summarized in Supplementary data online, *Table S5*. In the matched cohort, Kaplan–Meier curves and Cox models for the primary and secondary endpoints (*Figure 1C*), as well as logistic models for 1, 2, and 3 year incidence of those (*Table 2* and Supplementary data online, *Table S3*), confirmed significant association of TEER and favourable outcomes.

Finally, multivariable Cox proportional hazard models, adjusted for the same 31 covariates used to estimate the propensity score in the primary analysis, confirmed favourable association of TEER with the primary (HR 0.63, 95% CI 0.41–0.95, *P* = .028) and secondary endpoints (HR 0.59, 95% CI 0.35–0.98, *P* = .043) after adjustment for the 31 variables used to calculate the propensity score.

## Discussion

In this study, we demonstrated that patients with AFMR who underwent TEER had a lower risk of all-cause mortality and heart failure hospitalization compared with those who remained on conventional medical therapy. This association appeared more pronounced in patients whose residual MR severity was reduced to mild or less, as suggested by exploratory analyses, highlighting the crucial importance of effective MR reduction in the AFMR population (*Structured Graphical Abstract*). Although TEER for AFMR has recently been increasingly adopted in clinical practice, clear guideline recommendations have been lacking, and large-scale data have been scarce. Therefore, our findings provide important evidence supporting TEER as a new and promising therapeutic option for AFMR.

### Importance of AFMR in ageing societies

Atrial functional mitral regurgitation predominantly affects older adults, and its prevalence has been shown to rise with advancing age.^7^ The KUNIOMI registry, which examined an almost complete regional population of heart failure patients on a Japanese island, identified AFMR as the most common valvular disorder among individuals aged 75 years or older.^20^ In line with these observations, the mean age of AFMR patients in the present study was 80.1 years, highlighting that this condition primarily afflicts an elderly, high-risk cohort. In the global demographic shifts toward an ageing population, the demand for less invasive therapeutic interventions in such high-risk patients will be likely to continue to grow.

### Prognosis after TEER

Similar to other forms of functional MR, AFMR has been associated with unfavourable outcomes in proportion to increasing MR severity.^21^ Prior evidence further indicates that surgical correction of significant AFMR may improve clinical outcomes.^7^ Surgical mitral valve repair allows for concomitant treatment of associated lesions such as TR, and typically achieves a more durable reduction of MR than TEER, thereby positioning it as a potential first-line therapeutic strategy for severe AFMR.^3,10^ However, the advanced age, frailty, and malnutrition frequently observed in this population^9^ often elevate the procedural risk, rendering invasive surgery less feasible.

Transcatheter edge-to-edge repair is a less invasive procedure that has demonstrated substantial benefits in patients with VFMR, as confirmed by landmark RCTs such as the COAPT and RESHAPE-HF2 trials.^12,22^ Interestingly, MR severity in terms of effective regurgitant orifice area after overlap weighting in our cohort (0.26–0.29 mm^2^) was similar to that reported in the RESHAPE-HF2 trial (0.23 mm^2^). These findings have led to a rapid expansion of TEER use worldwide in the management of functional MR, and patients with AFMR who are at high surgical risk are frequently referred for this procedure. In the present analysis of the OCEAN-Mitral registry, among 2614 patients with functional MR who underwent clinically indicated TEER, 507 (19.4%) had AFMR. Despite its wide clinical application, several studies have noted that TEER in AFMR often results in greater residual MR and lower procedural success rates compared with that in VFMR.^23–25^ Furthermore, its prognostic impact in AFMR has not been thoroughly investigated.

To our knowledge, this is the first study to suggest a potential association between TEER and favourable prognosis in patients with minimal residual MR, based on both primary and exploratory analyses. These findings underscore the importance of achieving adequate MR reduction in the management of AFMR, although this should be considered an exploratory subgroup analysis. In the present analysis, TEER was associated with a reduced risk of all-cause mortality and heart failure hospitalization compared with medical therapy alone (HR 0.57–0.65). A 35%–43% reduction in hazard is comparable with those seen in the landmark COAPT trial (HR 0.62 for mortality)^12^ and are more favourable than those of cornerstone heart failure pharmacotherapies, such as sodium–glucose co-transporter 2 inhibitors and renin–angiotensin–aldosterone system inhibitors (typically HR 0.75–0.85).^26–28^ Although surgical treatment yielded an even lower HR of 0.39 in our previous study,^7^ this may reflect the additive benefit of concomitant interventions such as tricuspid valve surgery.

In absolute terms, the estimated incidence of the primary outcome was 21.7% at 1 year and 44.3% at 3 years in the medical therapy group, compared with 12.3% and 21.0%, respectively, in the TEER group. These differences became more pronounced over time, corresponding to an approximate absolute risk reduction of 9.4% at 1 year and 23.3% at 3 years. Such findings suggest that TEER may provide clinically meaningful benefit in this older and frail patient population with limited treatment options. Nevertheless, these results should be interpreted with caution due to the retrospective observational nature of the study, and prospective studies are warranted.

### Preferable target selection

Advanced age, female sex, and left atrial enlargement are known prognostic markers in heart failure.^29–31^ While our exploratory subgroup analyses showed no heterogeneity in the association between TEER and outcomes by age or sex, the relationship with left atrial size appeared more complex. A trend toward a stronger association between TEER and favourable outcomes was observed with increasing left atrial volume index (*P* for interaction = .089, *Figure 3*), and our spline analysis (see Supplementary data online, *Figure S4*) further suggested a non-linear association: the association appeared strongest within a certain range of left atrial enlargement, but diminished at the extremes. These findings suggest that while TEER may be associated with better outcomes in certain anatomical contexts, particularly within a specific range of left atrial size, a comprehensive and multidisciplinary treatment strategy remains essential. This includes optimal medical therapy, diligent management of comorbidities, and careful patient selection, considering factors such as the specific degree of left atrial enlargement, to optimize the use of TEER and its potential association with favourable outcomes in these high-risk individuals.

In this real-world analysis of consecutively enrolled patients undergoing TEER, 78.3% of those with AFMR exhibited mild or less residual MR, and 97.2% had moderate or less. The evolution of TEER devices in Japan and prudent patient selection may have contributed to these high success rates,^23,24,32^ which are likely to be associated with lower event rates in overall patients in the TEER group. Nevertheless, previous reports have identified anatomical characteristics, such as atriogenic tethering and a small leaflet-to-annulus index, as potential risk factors for procedural failure in TEER for AFMR.^23,24^ Although our study did not include detailed anatomical assessments, future investigations should determine which AFMR subgroups derive the greatest benefit from TEER by incorporating more comprehensive imaging and functional evaluations.

### Limitations

Several limitations of the study should be appropriately acknowledged. Although we applied multiple statistical adjustment methods and consistently observed lower event rates in the TEER group, the observational design does not exclude the possibility of unmeasured confounding or selection bias. In addition, we did not conduct a detailed anatomical evaluation specific to AFMR such as leaflet sizes and atriogenic tethering, so we were unable to determine which factors most strongly influence procedural success. Future investigations that employ randomized controlled designs and/or more comprehensive datasets are needed to clarify the causal relationship of TEER with favourable outcomes in AFMR and anatomical determinants of TEER success. Moreover, we did not assess the role of surgical intervention in the present analysis. Identifying which patients might benefit more from TEER or surgery and establishing an optimal treatment algorithm that incorporates both approaches will be important. Another limitation is the potential variability in echocardiographic assessment: findings were evaluated according to multiple criteria and quantification methods at each investigator’s discretion, which could introduce interobserver variability. Nonetheless, all transthoracic echocardiograms were prospectively obtained and evaluated by expert sonographers and cardiologists as part of a dedicated clinical study on MR assessment, ensuring a degree of consistency. Furthermore, the clear relationship between residual MR severity and prognosis suggests that any variability in grading did not compromise the significance of our findings, although future prospective studies would benefit from standardized core laboratory analyses. It should also be acknowledged that the exploratory finding suggesting an attenuation in the association between TEER and favourable outcomes at very large left atrial sizes (e.g. approximately beyond a left atrial volume index of 100 mL/m^2^) is likely to be based on relatively small numbers and should therefore be interpreted cautiously. Another limitation of this study is the lack of data on medication dosages, which were not incorporated in the analysis. Finally, although this was a multicentre investigation, it was conducted exclusively in Japan, which may limit the generalizability of these results to other populations.

## Conclusion

In conclusion, this cohort study demonstrated that TEER was associated with lower event rates in AFMR patients, proposing a valuable treatment alternative to conservative management in patients at high surgical risk. These findings contribute to advancements in AFMR treatment and lay the groundwork for future randomized trials to further elucidate the role of TEER in this context.

## Supplementary Material

ehaf511_Supplementary_Data

## Declarations

### Data Availability

The data underlying this study are not publicly available due to patient privacy concerns. However, de-identified data may be made available by the corresponding author upon reasonable request. Access to the data will require prior approval by the appropriate institutional review board or ethics committee, and may be subject to additional data use agreements.

### Pre-registered Clinical Trial Number

Both OCEAN-Mitral and REVEAL-AFMR registries were prospectively registered in the University Hospital Medical Information Network (UMIN) Clinical Trials Registry (UMIN000023653 and UMIN000046146) prior to patient enrolment.
