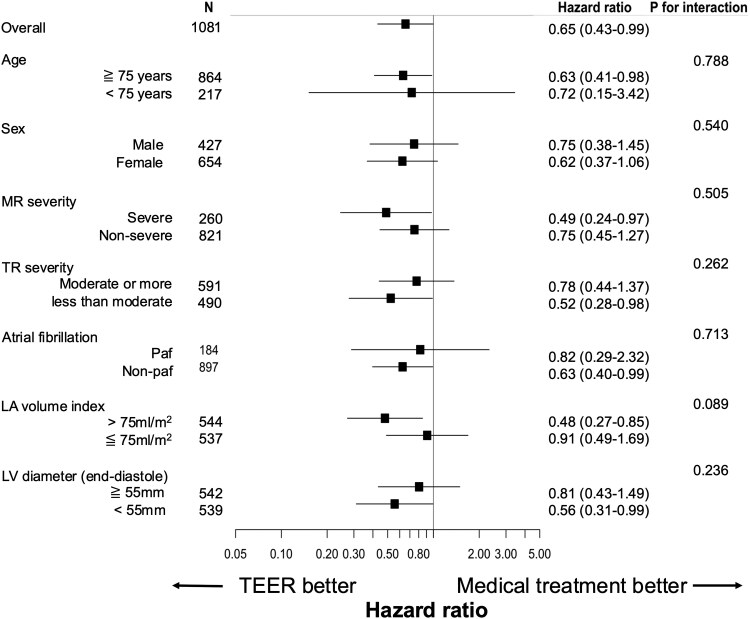# Correction to: Transcatheter edge-to-edge repair vs medical therapy in atrial functional mitral regurgitation: a propensity score-based comparison from the OCEAN-Mitral and REVEAL-AFMR registries

**DOI:** 10.1093/eurheartj/ehag190

**Published:** 2026-03-11

This is a correction to: Tomohiro Kaneko, Nobuyuki Kagiyama, Shinya Okazaki, Masashi Amano, Yukio Sato, Yohei Ohno, Masaru Obokata, Kimi Sato, Kojiro Morita, Shunsuke Kubo, Yuki Izumi, Masahiko Asami, Yusuke Enta, Shinichi Shirai, Masaki Izumo, Shingo Mizuno, Yusuke Watanabe, Makoto Amaki, Kazuhisa Kodama, Hisao Otsuki, Toru Naganuma, Hiroki Bota, Masahiro Yamawaki, Hiroshi Ueno, Gaku Nakazawa, Daisuke Hachinohe, Toshiaki Otsuka, Mike Saji, Masanori Yamamoto, Kentaro Hayashida, on behalf of the OCEAN-Mitral and the REVEAL-AFMR Investigators, Transcatheter edge-to-edge repair vs medical therapy in atrial functional mitral regurgitation: a propensity score-based comparison from the OCEAN-Mitral and REVEAL-AFMR registries, *European Heart Journal*, Volume 47, Issue 11, 14 March 2026, Pages 1304–1314, https://doi.org/10.1093/eurheartj/ehaf511

The forest plot in Figure 3 indicated a cutoff value of “55 mm” for LV diameter, this has been corrected to read “48 mm”.

Figure 3 should read:

**Figure:**
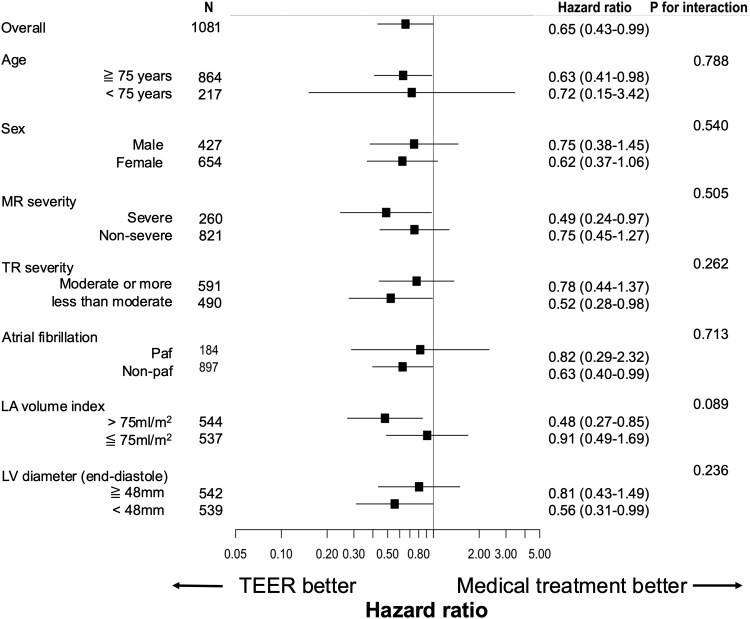


instead of:

**Figure:**