# Supplementary material for: Transcatheter edge-to-edge repair vs medical therapy in atrial functional mitral regurgitation: a propensity score-based comparison from the OCEAN-Mitral and REVEAL-AFMR registries
Source: Eur Heart J. 2025 Aug 1;47(11):1304–14. doi: 10.1093/eurheartj/ehaf511 (PMC13017546; doi:10.1093/eurheartj/ehaf511)
Supplement: ehaf511_Supplementary_Data [file ehaf511_Supplementary_Data.zip › RevealOcean Sup_figure_R2.doc]

## Supplementary Figure S1. Study flow chart

A schematic representation of the inclusion and exclusion criteria for TEER-treated and unoperated atrial functional mitral regurgitation patients from the OCEAN-Mitral and REVEAL-AFMR registries. The flow chart shows the final analyzed populations of 441 TEER-treated and 640 unoperated patients after applying exclusion criteria and handling missing data.

AFMR, atrial functional mitral regurgitation; DMR, degenerative mitral regurgitation; LA, left atrium; MVP, mitral valve plasty; MVR, mitral valve replacement; TEER, transcatheter edge-to-edge repair; VFMR, ventricular functional mitral regurgitation.

## Supplementary Figure S2. Kaplan–Meier curves for the primary and secondary endpoints in the unweighted cohort

Kaplan-Meier curves in the unweighted cohort for the composite endpoint of heart failure hospitalization or all-cause death (A) and all-cause death (B)

# Supplementary Figure S3: Changes in NYHA class from baseline to 1 year in the TEER and medication groups

In the weighted cohort, there was no significant difference in NYHA class between the two groups at baseline (p>0.999), whereas at the 1-year follow-up, NYHA class was significantly better in the TEER group (p<0.001).

NYHA, New York Heart Association; TEER, Transcatheter edge-to-edge repair

# Supplementary Figure S4: Restricted cubic spline analysis of hazard ratio by left atrial size

Non-linear associations of left atrial size and the HR of TEER versus medication for the primary outcome are summarized. As left atrial volume index (panel A) and diameter (panel B) increased, the HRs decreased up to a certain point. However, this trend plateaued, and the HR potentially increased, with marked left atrial enlargement (approximately beyond a left atrial volume index of 100 mL/m² or a diameter of 60 mm).
