# Supplementary material for: Transcatheter edge-to-edge repair vs medical therapy in atrial functional mitral regurgitation: a propensity score-based comparison from the OCEAN-Mitral and REVEAL-AFMR registries
Source: Eur Heart J. 2025 Aug 1;47(11):1304–14. doi: 10.1093/eurheartj/ehaf511 (PMC13017546; doi:10.1093/eurheartj/ehaf511)
Supplement: ehaf511_Supplementary_Data [file ehaf511_Supplementary_Data.zip › RevealOcean Sup_material_EHJ_R2.doc]

**Supplementary section of “Methods”**

**On-line Material**

# **Study design**

We utilized the following two registries to compare patients who underwent TEER with those who remained under medical treatment. The Optimized Catheter Valvular Intervention (OCEAN-Mitral) registry is an ongoing, prospective, multicenter registry involving 21 centers in Japan^1^. This registry, led by physicians to evaluate the safety and efficacy of TEER for MR, has enrolled consecutive adult patients (≥20 years old) with MR who underwent TEER from April 2018 to June 2023. TEER in this cohort was exclusively performed using the MitraClip device (Abbott, Santa Clara, CA, USA). The REVEAL-AFMR study was also a physician-initiated study to investigate the real-world characteristics and prognosis of patients with AFMR. This registry included consecutive adult patients (≥20 years old) with moderate or severe AFMR who underwent transthoracic echocardiography between January and December 2019 at 26 Japanese centers regardless of their treatment plan. The design, population, and primary results of these registries were previously published elsewhere^1,2^.

Since the OCEAN-Mitral and REVEAL-AFMR had different inclusion and exclusion criteria, we selected patients with moderate or severe AFMR who met the overlapping criteria of both registries to ensure a comparable study population. From each registry, we included patients with AFMR defined as MR in the absence of organic mitral valve disease, with normal left ventricular size and function (left ventricular end-diastolic volume <150ml, end-diastolic diameter <55mm, and ejection fraction ≥50%) and a dilated left atrium (left atrium volume index, ≥38 mL/m^2^ for men and ≥41 mL/m^2^ for women when available, or alternatively, left atrial diameter ≥40mm in men and ≥38mm in women) for the present analysis^2-4^. Patients who underwent surgical intervention were excluded from the REVEAL-AFMR registry. Furthermore, since there is partial overlap in participating centers between the two registries, some patients who underwent TEER enrolled in the REVEAL-AFMR registry might also be included in the OCEAN-Mitral registry. Therefore, 30 patients who underwent TEER in the REVEAL-AFMR registry were excluded to avoid potential duplication and associated bias. Supplementary Figure S1 shows the patient inclusion chart. Patients with missing data in the 31 baseline covariates used to estimate the propensity score (Supplemental Table 1) were excluded.

The study was conducted in compliance with the Declaration of Helsinki and the Japanese Ethical Guidelines for Medical and Health Research involving Human Subjects. The research protocol was approved by the Faculty Review Board of Keio University and Juntendo University, Japan, respectively. Both registries were prospectively registered in the University Hospital Medical Information Network (UMIN) Clinical Trials Registry (UMIN000023653 and UMIN000046146) prior to patient enrollment.

# **Endpoints**

The primary endpoints were composite of all-cause mortality and heart failure hospitalization, and the secondary endpoint was all-cause mortality. Prognostic data were collected from medical records and through telephone interviews when more than a year had elapsed since the patient's last follow-up. Heart failure hospitalization was defined as an unplanned admission for heart failure lasting ≥24 h, with new or worsening symptoms requiring initiation or intensification of therapy^5^. Acute Procedural Success was defined as successful implantation of the device with resulting MR severity of moderate or less on discharge echocardiogram without the occurrence of a mitral valve surgery (within 30 days) after the procedure^6^.

# **Propensity score-based overlap weighting**

Baseline characteristics differed substantially between the TEER and medication groups. To address this imbalance and to estimate the association between TEER and clinical outcomes, we employed several causal inference approaches.

As the primary analysis, we used propensity score-based overlap weighting, which assigns weights to each patient based on the probability of receiving the observed treatment given their baseline characteristics. This probability, propensity score, was estimated using multivariable logistic regression with 31 baseline covariates listed in Supplementary Table S1. Patients in the control group were weighted by each individual’s propensity score, while those in the treatment group by (1 – propensity score). This method creates a pseudo-population in which the distribution of baseline covariates is similar between the treatment groups, focusing on the region of overlap in propensity scores^7,8^. Given that the AFMR population is typically characterized by advanced age and frailty, estimation of the average treatment effect, rather than the average treatment effect on the treated, was deemed more appropriate to enhance the generalizability of the findings. Accordingly, a weighting approach was adopted. The primary analysis was conducted using overlap weighting, which allows inclusion of the entire study cohort while minimizing the influence of individuals with extreme propensity scores^8^. The standard errors and confidence intervals (CIs) for the estimates after propensity score weighting were calculated using a sandwich variance estimator. In the balanced cohort created by overlap weighting, we compared the incidence of the primary and secondary endpoints. After overlap weighting, Kaplan-Meier curves were constructed to visualize event rates in the TEER and the medication groups. Cox proportional hazards models were also constructed to assess the hazard ratio (HR) of the treatment with TEER for the endpoints. Similarly, logistic regression models were employed to assess the odds ratios (OR) of the treatment with 1-, 2-, and 3-year incidence of the endpoints. To identify subgroups in whom the association between TEER and clinical endpoints might be stronger, prespecified subgroup analyses for age, sex, MR severity, TR severity, paroxysmal atrial fibrillation, left atrial volume index, and left ventricular end-diastolic diameter were performed to test for interactions.

# **Sensitivity analysis**

## ***Propensity score-based analysis***

As a sensitivity analysis, we also performed inverse probability of treatment weighting (IPTW) using the same propensity score as in the overlap weighting. IPTW creates a pseudo-population in which treatment assignment is independent of observed covariates by weighting each patient by the inverse of their probability of receiving the treatment they actually received, i.e. ‘1 / propensity score’ for those in the TEER group and ‘1 / (1 – propensity score)’ for those in the medication group. To obtain the most accurate estimation of the average treatment effect without mechanically excluding the extremes of the propensity score distribution, we extracted the optimal subset sample using the ‘PStrim’ function from the ‘PSweight’ package version 1.2.0 in R.

As another sensitivity analysis, we performed 1:1 greedy propensity score matching using the same covariates as in the overlap weighting, with a caliper width of 0.2 standard deviations of the logit of the propensity score. This picks up the same number of patients from each group and creates pairs of patients with similar characteristics.

For patient populations created by these methods, we performed Kaplan-Meier analysis and Cox and logistic regression analyses as in the overlap weighting.

## ***Multivariable Cox proportional hazard models***

In addition to the propensity score-based analyses, we conducted a traditional multivariable Cox proportional hazard models to estimate the HRs of the treatment with TEER for the primary and secondary endpoints after adjusted for the same covariates as in the propensity score model.

# **Statistical analysis**

Categorical and continuous variables are presented as numbers (percentages) and means ± standard deviations or medians [interquartile ranges (IQR)], respectively. Differences in baseline characteristics between groups in the unweighted cohort were examined using Welch’s t-test, Wilcoxon rank-sum test, or Chi-squared test. To evaluate potential non-linear interactions between baseline left atrial size and treatment group on the primary endpoint, Cox proportional hazards models were utilized. These models included interaction terms between treatment group (TEER vs. medication) and restricted cubic spline functions with three knots representing left atrial volume index and diameter, respectively. The models allowed for estimation and visualization of the HR for the primary endpoint, comparing TEER to the medication reference group, across the continuous range of baseline left atrial sizes. All statistical analyses were performed using the R statistical software (version 4.3.2, R Foundation for Statistical Computing, Vienna, Austria). Statistical significance was defined as a two-sided p <0.05.

# **Reference**

1.	Saji M, Yamamoto M, Kubo S, Asami M, Enta Y, Shirai S*, et al.* Short-Term Outcomes Following Transcatheter Edge-to-Edge Repair: Insights From the OCEAN-Mitral Registry. *JACC Asia* 2023;**3**:766-773. doi: 10.1016/j.jacasi.2023.06.008

2.	Kagiyama N, Kaneko, T, Amano, M, Sato, Y, Ohno, Y, Obokata, M, Sato, K, Okada, T, Hoshino, N, Yamashita, K, Katsuta, Y, Izumi, Y, Ota, M, Mochizuki, Y, Sengoku, K, Sasaki, S, Nagura, F, Nomura, N, Nishikawa, R, Kato, N, Sakamoto, T, Eguchi, N, Senoo, M, Kitano, M, Takaya, Y, Saijo, Y, Tanaka, H, Nochioka, K, Omori, N, Tabata, M, Minamino, T, Hirose, N, Morita, K, Machino, T, Delgado, V, Abe Y. Clinical outcomes and impact of mitral valve surgery in atrial functional mitral regurgitation: the REVEAL-AFMR registry. *JAMA Netw Open*;**In press**. doi:

3.	Zoghbi WA, Levine RA, Flachskampf F, Grayburn P, Gillam L, Leipsic J*, et al.* Atrial Functional Mitral Regurgitation: A JACC: Cardiovascular Imaging Expert Panel Viewpoint. *JACC Cardiovasc Imaging* 2022;**15**:1870-1882. doi: 10.1016/j.jcmg.2022.08.016

4.	Farhan S, Silbiger JJ, Halperin JL, Zhang L, Dukkipati SR, Vogel B*, et al.* Pathophysiology, Echocardiographic Diagnosis, and Treatment of Atrial Functional Mitral Regurgitation: JACC State-of-the-Art Review. *J Am Coll Cardiol* 2022;**80**:2314-2330. doi: 10.1016/j.jacc.2022.09.046

5.	Abraham WT, Psotka MA, Fiuzat M, Filippatos G, Lindenfeld J, Mehran R*, et al.* Standardized definitions for evaluation of heart failure therapies: scientific expert panel from the Heart Failure Collaboratory and Academic Research Consortium. *Eur J Heart Fail* 2020;**22**:2175-2186. doi: 10.1002/ejhf.2018

6.	von Bardeleben RS, Rogers JH, Mahoney P, Price MJ, Denti P, Maisano F*, et al.* Real-World Outcomes of Fourth-Generation Mitral Transcatheter Repair: 30-Day Results From EXPAND G4. *JACC Cardiovasc Interv* 2023;**16**:1463-1473. doi: 10.1016/j.jcin.2023.05.013

7.	Cheng C, Li F, Thomas LE, Li FF. Addressing Extreme Propensity Scores in Estimating Counterfactual Survival Functions via the Overlap Weights. *Am J Epidemiol* 2022;**191**:1140-1151. doi: 10.1093/aje/kwac043

8.	Thomas LE, Li F, Pencina MJ. Overlap Weighting: A Propensity Score Method That Mimics Attributes of a Randomized Clinical Trial. *Jama* 2020;**323**:2417-2418. doi: 10.1001/jama.2020.7819
