# Supplementary material for: Transcatheter edge-to-edge repair vs medical therapy in atrial functional mitral regurgitation: a propensity score-based comparison from the OCEAN-Mitral and REVEAL-AFMR registries
Source: Eur Heart J. 2025 Aug 1;47(11):1304–14. doi: 10.1093/eurheartj/ehaf511 (PMC13017546; doi:10.1093/eurheartj/ehaf511)
Supplement: ehaf511_Supplementary_Data [file ehaf511_Supplementary_Data.zip › RevealOcean Sup_table_R2.doc]

## Supplementary Table S1. Variables for propensity score calculation

| Variables |  |
| --- | --- |
| sex | prescription of loop diuretics |
| age | prescription of MRA |
| BSA | prescription of beta-blocker |
| NYHA | prescription of tolvaptan |
| NYHA(Ⅲ/Ⅳ) | hemoglobin |
| history of hypertension | eGFR |
| history of diabetes mellites | serum creatinine |
| paroxysmal atrial fibrillation | LA volume index |
| chronic atrial fibrillation | LVEF |
| prior HF hospitalization | LV end-diastolic dimension |
| history of smoking | LV end-systolic dimension |
| history of COPD | MR severity |
| history of cancer | severe MR |
| prior symptomatic stroke | TR severity |
| history of coronary artery disease | severe TR |
| significant AS |  |

AS, aortic stenosis; BSA, body surface area; COPD, chronic obstructive pulmonary disease; eGFR, estimated glomerular filtration rate; HF, heart failure; LA, left atrium; LV, left ventricle; LVEF, left ventricular ejection fraction; MR, mitral regurgitation; MRA, mineralocorticoid receptor antagonist; NYHA, New York Heart Association; TR, tricuspid regurgitation.

## Supplementary Table S2: Baseline medical therapy in the TEER and medication groups

|  |  | Original baseline characteristics | | | Overlap-weighted characteristics | | |
| --- | --- | --- | --- | --- | --- | --- | --- |
|  | Missing (%) | TEER | Medication | p-value | TEER | Medication | SMD |
| ***Prescription*** |  |  |  |  |  |  |  |
| Loop diuretics | 0 (0%) | 371 (84.1%) | 323 (50.5%) | <0.001 | 73.4% | 73.4% | 0 |
| Tolvaptan | 0 (0%) | 175 (39.7%) | 56 (8.8%) | <0.001 | 21.5% | 21.5% | 0 |
| Beta-blocker | 0 (0%) | 308 (69.8%) | 318 (49.7%) | <0.001 | 64.9% | 64.9% | 0 |
| MRA | 0 (0%) | 217 (49.2%) | 149 (23.3%) | <0.001 | 42.6% | 42.6% | 0 |
| ACE-I/ARB | 5 (0.5%) | 221 (50.1%) | 268 (41.9%) | 0.005 | 49.8% | 43.0% | 0.136 |
| SGLT-2 inhibitor | 0 (0%) | 63 (14.3%) | 10 (1.6%) | <0.001 | 16.2% | 1.2% | 0.553 |

ACE-I, angiotensin converting enzyme inhibitor; ARB, angiotensin receptor blocker; MRA, mineralocorticoid receptor antagonist; SGLT-2, sodium-glucose cotransporter-2; SMD, standard mean difference.

## Supplementary Table S3. Regression analysis for the secondary endpoints at 1, 2, and 3 years

|  |  | **Estimated incidence of**  **the secondary endpoint, %** | | **OR** | **95% CI** | **p-value** |
| --- | --- | --- | --- | --- | --- | --- |
|  |  | TEER | Medication |  |  |  |
| **Overlap weighting** | 1 year | 5.0 | 12.3 | 0.38 | [0.17 - 0.86] | 0.020 |
|  | 2 years | 9.0 | 22.8 | 0.34 | [0.17 - 0.65] | 0.001 |
|  | 3 years | 10.9 | 28.6 | 0.31 | [0.17 - 0.56] | <0.001 |
| **IPTW** | 1 year | 4.5 | 12.7 | 0.32 | [0.12 - 0.85] | 0.022 |
|  | 2 years | 8.6 | 20.4 | 0.37 | [0.17 - 0.79] | 0.010 |
|  | 3 years | 10.5 | 25.5 | 0.34 | [0.17 - 0.68] | 0.002 |
| **PS matching** | 1 year | 3.8 | 13.5 | 0.26 | [0.07 - 0.75] | 0.007 |
|  | 2 years | 8.7 | 27.9 | 0.25 | [0.10 - 0.53] | 0.001 |
|  | 3 years | 12.5 | 34.6 | 0.27 | [0.13 - 0.54] | <0.001 |
| **Unadjusted analysis** | 1 year | 10.7 | 8.0 | 1.38 | [0.91 - 2.09] | 0.132 |
|  | 2 years | 16.6 | 14.1 | 1.21 | [0.86 - 1.69] | 0.261 |
|  | 3 years | 20.2 | 17.3 | 1.20 | [0.88 - 1.64] | 0.238 |

CI, confidence interval; IPTW, inverse probability of treatment weighting; OR, odds ratio; PS, propensity score; TEER, transcatheter edge-to-edge repair

## Supplementary Table S4. Characteristics after IPTW

| Variable | TEER | Medication | SMD |
| --- | --- | --- | --- |
| N | 158 | 173 |  |
| Male | 34.3% | 32.8% | 0.032 |
| Age, years | 81.2 | 81.2 | 0.005 |
| NYHA (III or IV) | 30.4% | 25.6% | 0.108 |
| Hypertension | 76.5% | 73.7% | 0.064 |
| Permanent atrial fibrillation | 60.9% | 64.2% | 0.067 |
| Coronary artery disease | 20.0% | 18.6% | 0.037 |
| Loop diuretics | 75.9% | 74.8% | 0.026 |
| Moderate-severe MR | 33.7% | 34.3% | 0.013 |
| Severe MR | 24.8% | 25.5% | 0.016 |
| LVDd, mm | 46.7 | 47.0 | 0.053 |
| LVDs, mm | 31.9 | 30.8 | 0.063 |
| LAVI, ml/m^2^ | 93.4 | 91.7 | 0.034 |
| LVEF, % | 61.0 | 60.9 | 0.027 |
| Hemoglobin, g/dL | 11.2 | 11.4 | 0.086 |
| COPD | 7.2% | 4.9% | 0.086 |
| Diabetes mellites | 22.5% | 21.2% | 0.030 |
| eGFR, mL/min/1.73m^2^ | 48.1 | 48.6 | 0.022 |
| Prior symptomatic stroke | 6.1% | 5.5% | 0.027 |
| Cancer | 14.4% | 16.1% | 0.048 |
| Moderate or severe AS | 6.1% | 8.8% | 0.104 |
| Moderate TR | 33.5% | 33.7% | 0.004 |
| Moderate-severe TR | 12.2% | 12.5% | 0.009 |
| Severe TR | 5.4% | 7.0% | 0.064 |
| Beta-blocker | 66.0% | 63.3% | 0.056 |
| Tolvaptan | 23.4% | 25.7% | 0.053 |
| Paroxysmal atrial fibrillation | 18.1% | 15.1% | 0.080 |
| Prior HF admission | 61.8% | 62.5% | 0.015 |
| MRA | 48.5% | 45.1% | 0.066 |
| Body surface area, m^2^ | 1.47 | 1.46 | 0.020 |
| Creatinine, mg/dL | 1.33 | 1.36 | 0.026 |
| History of smoking | 26.6% | 25.0% | 0.037 |

AS, aortic stenosis; COPD, chronic obstructive pulmonary disease; eGFR, estimated glomerular filtration rate; HF, heart failure; LAVI, left atrial volume index; LVDd, left ventricular end-diastolic diameter; LVDs, left ventricular end-systolic diameter; LVEF, left ventricular ejection fraction; MR, mitral regurgitation; MRA, mineralocorticoid receptor antagonist; NYHA, New York Heart Association; TEER, transcatheter edge-to-edge repair; TR, tricuspid regurgitation.

## Supplementary Table S5. Characteristics of patients after propensity score matching

| Variable | TEER | Medication | SMD |
| --- | --- | --- | --- |
| N | 104 | 104 |  |
| Male | 33.7% | 36.5% | 0.059 |
| Age, years | 82.5 | 81.6 | 0.138 |
| NYHA (III or IV) | 28.8% | 29.8% | 0.019 |
| Hypertension | 77.9% | 75.0% | 0.064 |
| Permanent atrial fibrillation | 62.5% | 62.5% | 0.000 |
| Coronary artery disease | 17.3% | 18.3% | 0.023 |
| Loop diuretics | 74.0% | 71.2% | 0.026 |
| Moderate-severe MR | 32.7% | 32.7% | 0.000 |
| Severe MR | 22.1% | 22.1% | 0.000 |
| LVDd, mm | 46.5 | 47.3 | 0.114 |
| LVDs, mm | 31.0 | 31.1 | 0.024 |
| LAVI, ml/m^2^ | 90.1 | 94.9 | 0.063 |
| LVEF, % | 60.3 | 60.9 | 0.088 |
| Hemoglobin, g/dL | 11.3 | 11.3 | 0.026 |
| COPD | 5.8% | 4.8% | 0.035 |
| Diabetes mellites | 20.2% | 14.4% | 0.160 |
| eGFR, mL/min/1.73m^2^ | 47.1 | 48.2 | 0.058 |
| Prior symptomatic stroke | 5.8% | 7.7% | 0.058 |
| Cancer | 13.5% | 17.0% | 0.080 |
| Moderate or severe AS | 7.7% | 8.7% | 0.049 |
| Moderate TR | 25.0% | 37.5% | 0.039 |
| Moderate-severe TR | 14.4% | 10.6% | 0.039 |
| Severe TR | 6.7% | 7.7% | 0.055 |
| Beta-blocker | 69.2% | 60.6% | 0.189 |
| Tolvaptan | 20.2% | 18.3% | 0.039 |
| Paroxysmal atrial fibrillation | 15.4% | 16.3% | 0.025 |
| Prior HF admission | 59.6% | 53.8% | 0.144 |
| MRA | 41.3% | 44.2% | 0.058 |
| Body surface area, m^2^ | 1.46 | 1.46 | 0.013 |
| Creatinine, mg/dL | 1.36 | 1.36 | 0.008 |
| History of smoking | 25.0% | 32.7% | 0.170 |

AS, aortic stenosis; COPD, chronic obstructive pulmonary disease; eGFR, estimated glomerular filtration rate; HF, heart failure; LAVI, left atrial volume index; LVDd, left ventricular end-diastolic diameter; LVDs, left ventricular end-systolic diameter; LVEF, left ventricular ejection fraction; MR, mitral regurgitation; MRA, mineralocorticoid receptor antagonist; NYHA, New York Heart Association; TEER, transcatheter edge-to-edge repair; TR, tricuspid regurgitation.
